# Development and Optimization of Expression, Purification, and ATPase Assay of KaiC for Medium-Throughput Screening of Circadian Clock Mutants in Cyanobacteria

**DOI:** 10.3390/ijms20112789

**Published:** 2019-06-07

**Authors:** Dongyan Ouyang, Yoshihiko Furuike, Atsushi Mukaiyama, Kumiko Ito-Miwa, Takao Kondo, Shuji Akiyama

**Affiliations:** 1 Research Center of Integrative Molecular Systems (CIMoS), Institute for Molecular Science, National Institute for Natural Sciences, 38 Nishigo-Naka, Myodaiji, Okazaki 444-8585, Japan; ouyangdy@ims.ac.jp (D.O.); furuike@ims.ac.jp (Y.F.); amukai@ims.ac.jp (A.M.); 2 Department of Functional Molecular Science, SOKENDAI (The Graduate University for Advanced Studies), 38 Nishigo-Naka, Myodaiji, Okazaki 444-8585, Japan; 3 Division of Biological Science, Graduate School of Science and Institute for Advanced Research, Nagoya University; Furo-cho, Chikusa-ku, Nagoya 464-8602, Japan; miwa@bio.nagoya-u.ac.jp (K.I.-M.); kondo@bio.nagoya-u.ac.jp (T.K.)

**Keywords:** circadian clock, cyanobacteria, KaiC, scanning mutagenesis, ATPase activity, temperature compensation, phosphorylation cycle

## Abstract

The slow but temperature-insensitive adenosine triphosphate (ATP) hydrolysis reaction in KaiC is considered as one of the factors determining the temperature-compensated period length of the cyanobacterial circadian clock system. Structural units responsible for this low but temperature-compensated ATPase have remained unclear. Although whole-KaiC scanning mutagenesis can be a promising experimental strategy, producing KaiC mutants and assaying those ATPase activities consume considerable time and effort. To overcome these bottlenecks for in vitro screening, we optimized protocols for expressing and purifying the KaiC mutants and then designed a high-performance liquid chromatography system equipped with a multi-channel high-precision temperature controller to assay the ATPase activity of multiple KaiC mutants simultaneously at different temperatures. Through the present protocol, the time required for one KaiC mutant is reduced by approximately 80% (six-fold throughput) relative to the conventional protocol with reasonable reproducibility. For validation purposes, we picked up three representatives from 86 alanine-scanning KaiC mutants preliminarily investigated thus far and characterized those clock functions in detail.

## 1. Introduction

Circadian clocks are self-sustained oscillatory systems allowing organisms to anticipate and adapt to the daily environmental changes resulting from the Earth’s rotation [1]. This time-measuring machinery has been ubiquitously observed from eukaryotes [2] to prokaryotes [3] and is known to possess three remarkable characteristics [4]—autonomous oscillation with a period of approximately 24 h, temperature independency of the period length (temperature compensation), and entrainability by external perturbations. Principal constituents of the circadian clock systems are genes responsible for the clock functions (clock genes) and their translational products (clock proteins). Circadian rhythms arising as consequences of negative feedback regulations of clock gene transcriptions by the clock proteins are called transcriptional and translational oscillations (TTO) [5], while those driven solely by the clock proteins are called post-translational oscillations (PTO) [6].

The cyanobacterium *Synechococcus elongatus* PCC7942 (*S. elongatus*) is one of the organisms harboring both TTO [7] and PTO [8]. *S. elongatus* PTO can be reconstructed in vitro as a phosphorylation cycle of KaiC by mixing KaiC with the other two Kai proteins, KaiA and KaiB, in the presence of adenosine triphosphate (ATP) [9]. KaiC reveals rhythmic activation and inactivation of auto-kinase [9], auto-phosphatase [9], and ATPase [10] activities to affect repeated assembly and disassembly of Kai protein complexes [11,12]. KaiC ATPase is of particular interest here, as its activity is extremely low (~12 ATP d^-1^) [10], temperature compensated (*Q*_10_ = 1.0) [10], and finely correlated to the frequencies of TTO as well as PTO [10,13]. These unique properties inspire development of an ATPase-based screening for KaiC clock mutants giving short, long, and/or temperature-dependent periods, as one of the effective methods to uncover origins of the temperature-compensated circadian period in the *S. elongatus* clock system.

In this paper, we describe the current design of our in vitro ATPase-based screening system and discuss its performance using the single-alanine substitutions (alanine scanning) library of KaiC. The alanine scanning itself is an established means often used to test hypothesis on structure–function relationships in proteins [14,15]; however, its application to the ATPase-based screening is not straightforward, because several experimental steps, such as mutant expression, purification, and ATPase assay, limit its throughput.

To improve the efficiency of the in vitro screening, we replaced a glutathione *S*-transferase (GST)-tag fused to KaiC by a hexa-histidine (His)-tag and then optimized procedures to express and purify His-tagged KaiC. Furthermore, a new high-performance liquid chromatography (HPLC) system with a four-channel temperature controller was designed to determine temperature dependencies of the ATPase activities for multiple KaiC mutants simultaneously. These improvements together with several other optimizations reduced approximately 80% of the time costs associated with the overall screening process. Thus far, 16.5% of the alanine scanning KaiC mutants (86 of 519 amino acid residues) have been preliminary expressed, purified, and analyzed with the improved throughput. For validation purposes, three representative KaiC mutants revealing modulated ATPase activity and/or its temperature dependency were subjected to in vivo TTO and in vitro PTO assays. We discuss future perspectives of the ATPase-based in vitro screening on the basis of advantages and disadvantages of the present method.

## 2. Results

### 2.1. Optimization of the Expression and Purification of KaiC

**Figure 1:**
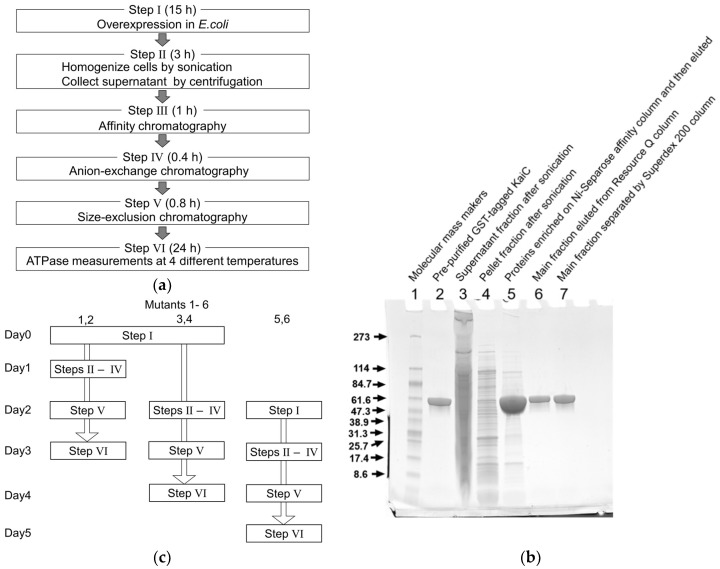
Optimized schemes for expression, purification, and ATPase-assay of hexa-histidine (His)-tagged KaiC. (**a**) Overview of the protocol. (**b**) Sodium dodecyl sulfate-polyacrylamide gel electrophoresis (SDS-PAGE) analysis of His-tagged KaiC during its purification. Lane 1, molecular mass makers; lane 2, the pre-purified glutathione *S*-transferase (GST)-tagged KaiC; lane 3, the supernatant fraction after sonication; lane 4, the pellet fraction after sonication; lane 5, the proteins enriched on Ni-Separose affinity column and then eluted; lane 6; the main fraction eluted from Resource Q column; lane 7, the main fraction separated by Superdex 200 column. (**c**) Pseudo-parallel treatment of three sets of two KaiC mutants to achieve six KaiC mutants per week.

In an expression vector (pGEX-6P-1 *kaiC*) originally constructed by Iwasaki et al. [16], the C-terminus of GST is fused to the N-terminus of *S. elongatus* KaiC consisting of 519 amino acid residues. KaiC is thus expressed as an N-terminally GST-tagged form and has to be treated with proteinases for a long time to cleave the GST-tag off. To skip this time-consuming treatment, we constructed an expression vector encoding KaiC in a C-terminally His-tagged form (pET-3a *kaiC*). In this study, *Escherichia coli*(*E. coli*)BL21(DE3)pLysE was used as a host for His-tagged KaiC over-expression (Step I in Figure 1a), as it allowed more expression in a culture volume of 400 mL within a shorter culture time than BL21(DE3) strain (see details in Materials and Methods).

In the purification process (Step II in Figure 1a), His-tagged KaiC was first extracted by suspending cell pellets in 25 mL of a buffer containing 20 mM Tris/HCl, 0.15 M NaCl, 0.5 mM ethylenediaminetetraacetic acid (EDTA), 1 mM ATP, 5 mM MgCl_2_, 1 mM dithiothreitol (DTT), and 40 U/mL benzonase at pH 8.0. After 1 h stirring, the cells were then crushed by sonication. The extraction of His-tagged KaiC was further promoted by adding tritonX-100 solution to a final concentration of 1% and by stirring for another 1 h.

A supernatant fraction separated by centrifugation (lane 3 in Figure 1b) was applied to a free-packed Ni-Sepharose affinity column (bead volume of 2 mL) equilibrated with a binding buffer containing 20 mM Tris/HCl, 500 mM NaCl, 5 mM MgCl_2_, 1 mM ATP, and 130 mM imidazole at pH 7.4 (Step III in Figure 1a). The column after loading the sample was washed with 25 mL of the binding buffer. His-tagged KaiC was eluted with an elution buffer containing 20 mM Tris/HCl, 500 mM NaCl, 5 mM MgCl_2_, 1 mM ATP, and 480 mM imidazole at pH 7.4. Immediately after the elution, the fractions containing imidazole were subjected to a PD-10 desalting column (GE Healthcare) equilibrated with 20 mM Tris/HCl, 0.1 M NaCl, 0.5 mM EDTA, 1 mM ATP, 5 mM MgCl_2_, and 1 mM DTT at pH 8.0 to remove imidazole because of a poor compatibility between imidazole and His-tagged KaiC. All of the washing, eluting, and buffer exchanging steps were thus conducted quickly to minimize the exposure of His-tagged KaiC to imidazole to prevent the sample from ending with a cloudy solution, or, even worse, precipitation.

Impurities remaining even after Ni-Sepharose affinity chromatography (lane 5 in Figure 1b) were separated through two-step chromatographic purification procedure (Steps IV and V in Figure 1a). The desalted sample was first loaded to an anion-exchange column (Resource Q) equilibrated with 20 mM Tris/HCl, 0.1 M NaCl, 0.5 mM EDTA, 1 mM ATP, 5 mM MgCl_2_, and 1 mM DTT at pH 8.0, and the resultant main fractions (lane 6 in Figure 1b) were subjected to a size-exclusion column (Superdex 200) equilibrated with 20 mM Tris/HCl, 0.15 M NaCl, 0.5 mM EDTA, 1 mM ATP, 5 mM MgCl_2_, and 1 mM DTT at pH 8.0 to achieve high purities of more than 95% (lane 7 in Figure 1b). According to the results of more than 86 mutants tried thus far, the final yield was maintained at least 0.6 mg per 400 mL of terrific broth medium for each sample. This was nearly the amount (~0.7 mg) of His-tagged KaiC sufficient to measure its ATPase activity at four different temperatures (Step VI in Figure 1a) [10].

### 2.2. Development of the HPLC System Equipped with a Multi-Channel High-Precision Temperature Controller

ATPases are enzymes catalyzing the hydrolysis of ATP into adenosine diphosphate (ADP) and phosphate (Pi). Time courses of ADP production have been measured thus far using conventional columns and HPLC to evaluate the steady-state ATPase activity of KaiC [10,17]. Because most of the commercial HPLCs are equipped with just one sample plate thermostated at one particular temperature, multiple rounds of additional experiments at different temperatures were required to estimate *Q*_10_ values.

**Figure 2:**
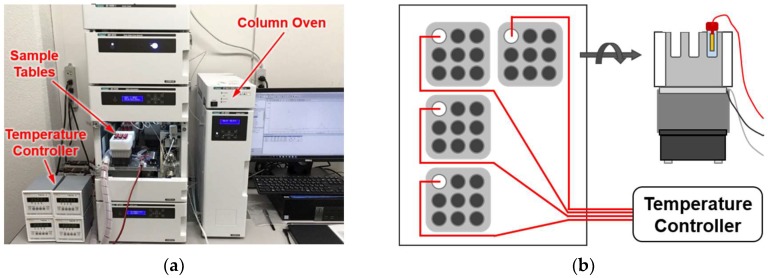
High-performance liquid chromatography (HPLC) system equipped with four-channel temperature controller. (**a**) Photograph of an HPLC equipped with four sample tables controlled independently at different temperatures. (**b**) Schematic top and side views of the four sample tables, each carrying nine sample holders thermostated by a Peltier device.

To save this time cost, we developed a set of four sample tables, each thermostated independently at different temperatures, and installed it into a commercial HPLC (Figure 2a). Nine sample holders were fabricated on each sample table. A Pt100 sensor was directly inserted into a water-filled reference vial placed at one of the nine sample holders so that the samples were maintained at the target temperature with a precision of ± 0.01 °C (Figure 2b). This standard setup allowed the simultaneous analysis of (maximally) eight different samples/references at four different temperatures, saving the time required for reasonable estimations of the *Q*_10_ values while maintaining experimental quality.

### 2.3. Throughput, Performance, and Reproducibility of the Protocol

Our experiences working along with the present protocol suggest that a better throughput is expected by expressing, purifying, and analyzing three sets of two KaiC mutants in a pseudo-parallel manner (Figure 1c). One factor limiting further parallel processing by one worker is the requirement for His-tagged KaiC to be separated immediately from imidazole using the open column. The other is a preventive measure against unexpected actions of mistaking one sample for another. Even with these practical limitations, our method permits a six-fold throughput (six KaiC mutants per week) for analyzing the ATPase activities at four different temperatures as compared to the conventional analysis of GST-tagged KaiC with ordinary HPLC (just under one KaiC mutant per week) [10,17].

**Figure 3:**
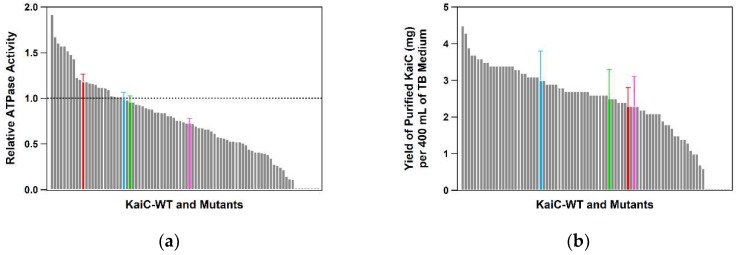
Preliminary dataset for 86 KaiC mutants. (**a**) Distribution of the relative ATPase activities. (**b**) Distribution of the final yields (per 400 mL of terrific broth medium) of His-tagged KaiC. Mean values for KaiC-WT, KaiC-P37A, KaiC-T228A, and KaiC-C306A are represented as blue, green, magenta, and red bars, respectively, with standard deviation from three independent experiments.

To assess the impact of single-alanine substitutions, we estimated a distribution of relative ATPase activities (Figure 3a) using a preliminary dataset for 86 KaiC mutants prepared/analyzed along with the present protocol. As expected, the distribution included the KaiC mutants carrying activated and inactivated ATPases.

Reproducibility is another important factor required for screening processes. For validation purposes, we chose three KaiC mutants (KaiC-P37A, KaiC-T228A, and KaiC-C306A) from the preliminary dataset and estimated those ATPase activities using three independent preparations. Total errors (SD) arising during the expression, the purification, and the ATPase assay were 7~14% (Table 1), well below the difference in the ATPase activities between the hyper-activated and the hyper-inactivated mutants (Figure 3a). Furthermore, most of the 86 KaiC mutants could be expressed and purified successfully through the optimized protocol except for eight mutants with poor expression (Figure 1a), yielding, on average, 2.4 ± 1.1 mg (per 400 mL of terrific broth medium) of His-tagged KaiC mutants sufficient to assay both ATPase activity and phosphorylation cycle (Figure 3b). These observations indicate that the combined usage of the present protocol and the developed HPLC system provides the opportunity of screening the ATPase mutants of KaiC with reasonable throughput, performance, and reproducibility.

### 2.4. Detailed Characterizations of Representative ATPase Mutants of KaiC

**Figure 4:**
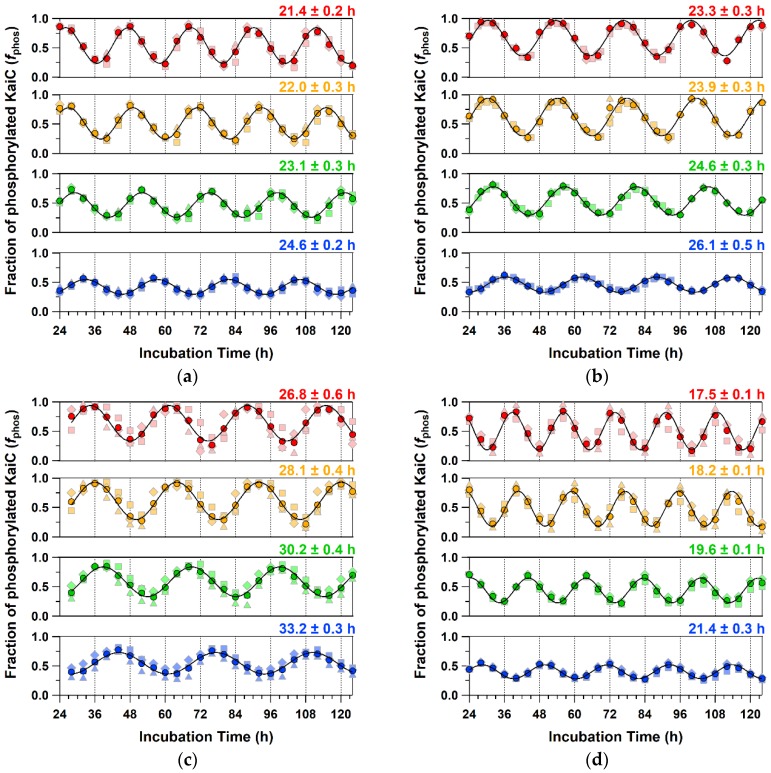
Phosphorylation/dephosphorylation cycles of (**a**) KaiC-WT, (**b**) KaiC-P37A, (**c**) KaiC-T228A, and (**d**) KaiC-C306A at different temperatures. Blue, green, orange, and red markers correspond to the fractions of phosphorylated KaiC (*f*_phos_) at 25, 30, 35, and 40 °C, respectively. Dark-colored circles correspond to the mean from independent preparations and measurements (pale-colored squares, triangles, and diamonds). Thin lines represent the fitting of the mean to Equation (1) described in Materials and Methods.

**Figure 5:**
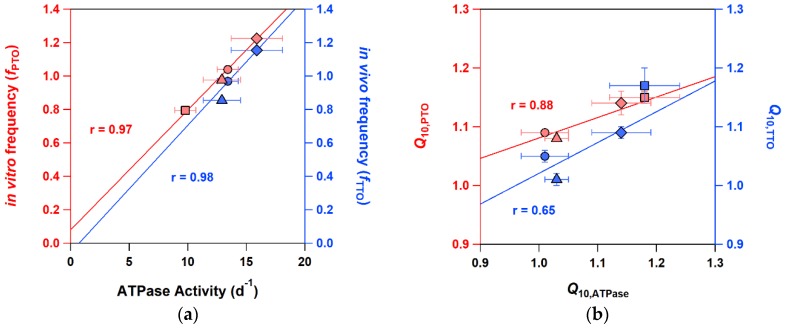
Cross-correlational plots of (**a**) in vivo frequency of bioluminescence rhythms (*f*_TTO_), in vitro frequency of KaiC phosphorylation cycles (*f*_PTO_), and in vitro ATPase activity of KaiC alone at 30 °C, and of (**b**) the *Q*_10_ values for bioluminescence rhythms (*Q*_10,TTO_, 25~29 °C), KaiC phosphorylation cycles (*Q*_10,PTO_, 25~35 °C), and in vitro ATPase activity of KaiC alone (*Q*_10,ATPase_, 25~35 °C). Filled circles, triangles, squares, and diamonds correspond to KaiC-WT, KaiC-P37A, KaiC-T228A, and KaiC-C306A, respectively. Red and blue markers correspond to in vitro (PTO) and in vivo (TTO) data, respectively. Thick lines represent linear fits to the data with correlation coefficients (r).

To assess the mutation–phenotype relationships, we reconstructed PTO by mixing each of the three KaiC mutants with both KaiA and KaiB and characterized their oscillatory properties (Figure 4). The PTO for KaiC-C306A revealed a short period of 19.6 ± 0.1 h at 30 °C (Figure 4d), while those of KaiC-P37A (Figure 4b) and KaiC-T228A (Figure 4c) showed evidently long periods of 24.6 ± 0.3 and 30.2 ± 0.4 h, respectively. The order of oscillation frequency [24 × (period length)^−1^] was KaiC-C306A (1.22 d^−1^) > KaiC-WT (1.04 d^−1^) > KaiC-P37A (0.98 d^−1^) > KaiC-T228A (0.79 d^−1^), matching that of the ATPase activity of the individual KaiC mutants (Table 1, Figure 5a). The *Q*_10_ values of the ATPase activity for KaiC-C306A (1.14 ± 0.05) and KaiC-T228A (1.18 ± 0.06) were larger than that for KaiC-WT (1.01 ± 0.04). Consistent with this observation, the period length of PTO for KaiC-C306A was gradually but evidently shortened from 21.4 to 17.5 h as the temperature increased from 25 to 40 °C (Figure 4d). KaiC-T228A revealed similar or more pronounced shortening of the period length from 33.2 h at 25°C to 26.8 h at 35°C (Figure 4c). These observations support the effectiveness of in vitro ATPase-based screening in identifying KaiC mutations affecting the period length and/or its temperature compensation in PTO.

**Figure 6:**
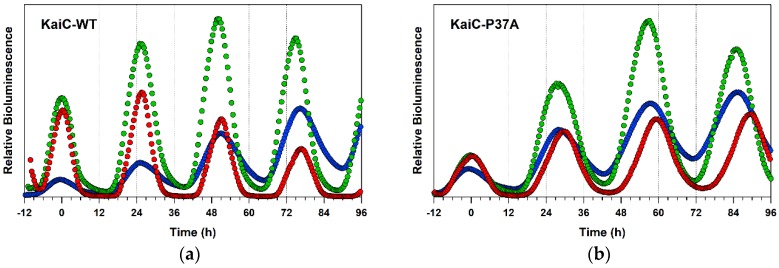

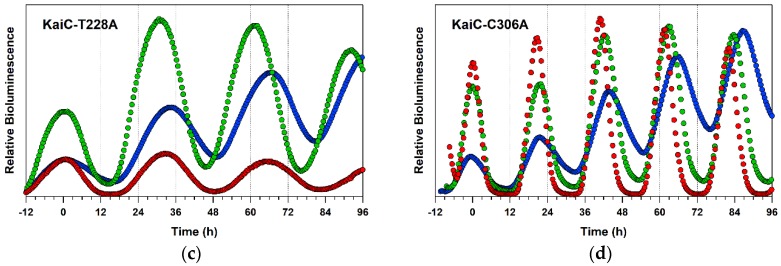
In vivo bioluminescence rhythms of (**a**) KaiC-WT, (**b**) KaiC-P37A, (**c**) KaiC-T228A, and (**d**) KaiC-C306A at different temperatures. Blue, green, and red circles correspond to profiles recorded at 25, 29, and 35 °C, respectively. For clarity of comparison, each profile is shifted horizontally so that the peak matches 0 h.

To examine the correspondence to in vivo clock properties, we constructed a bioluminescent cyanobacterial reporter strain [7,9] carrying each of the KaiC-P37A, the KaiC-T228A, and the KaiC-C306A substitutions (Figure 6). As shown in Table 1, the period length of the bioluminescence rhythm was nearly matched to that of in vitro PTO as was observed previously [9]. Interestingly, the bioluminescence rhythm of the strain carrying the KaiC-C306A mutation was gradually accelerated in a temperature-dependent manner (Figure 6d). The *Q*_10_ value of the KaiC-C306A strain was estimated using the profiles taken at 25 and 29 °C to be 1.09 ± 0.01 and was slightly smaller than that (1.14 ± 0.02) of in vitro PTO (t-test: *p* = 0.027). It should be noted, however, that the *Q*_10_ value of KaiC-C306A was always larger than that of KaiC-WT in both cases of PTO (t-test: *p* = 0.031) and TTO (t-test: *p* = 0.006) (Table 1). Although the period length for the KaiC-T228A strain was unexpectedly prolonged from 30.2 ± 0.2 to 32.9 ± 0.1 by raising the temperature from 29 to 35 °C (Figure 6c), its *Q*_10_ value (1.17 ± 0.03) calculated using the period lengths at 25 and 29 °C was consistent with the in vitro result (*Q*_10_ = 1.15 ± 0.01) (t-test: *p* = 0.321) (Table 1). A similar tendency was also observed for KaiC-P37A for some unknown reasons (Figure 6b). While the consistency of the *Q*_10_ values between PTO and TTO can be influenced by the temperature range used for evaluation (Table 1), in vitro ATPase-based screening is, in principle, beneficial for studying both PTO and TTO.

## 3. Discussion

In this paper, the effectiveness of in vitro ATPase-based screening was evaluated in terms of its throughput, performance, and reproducibility. The present throughput of six KaiC mutants per week allows us to estimate the time required for completing the whole-KaiC scanning mutagenesis to be approximately 1.6 years. The in vivo bioluminescence screening system [7,9] may provide an equivalent or even higher throughput given the availability of the multiple apparatuses placed in independently temperature-controlled rooms. The developed set of four sample tables is compact enough to be installed to the sample compartment of ordinary HPLC systems, providing a moderate-throughput screening of the KaiC clock mutants with a reasonable setup cost and space.

The results of the three representative mutants support the performance of our ATPase-based screening system. As shown in Table 1 and Figure 5, the KaiC mutations showing lower and higher ATPase activities were demonstrated to give longer and shorter periods, respectively, in both PTO and TTO. This correspondence means that the ATPase activity is the excellent measure of the period length in the cyanobacterial circadian clock.

It deserves to be noted that there was an effect of KaiA and/or KaiB on the *Q*_10_ values in PTO. As shown in Table 1, the *Q*_10_ values of PTO (1.09 ± 0.00) for KaiC-WT were somewhat larger than that of ATPase (1.01 ± 0.04) (t-test: *p* = 0.062). The similar tendency was also confirmed in KaiC-P37A (t-test: *p* = 0.067). These observations may be explained by the fact that the *Q*_10_ value of ATPase becomes slightly affected by temperature when both KaiA and KaiB coexist [10]. The less significant correlation of *Q*_10,ATPase_ with *Q*_10,TTO_ than with *Q*_10,PTO_ (Figure 5b) may suggest a pronounced KaiA/KaiB-related effect in vivo. It is surprising that, given this potential KaiA/KaiB-related effect, our system is sensitive enough to isolate the mutations (KaiC-T228A and KaiC-C306A), resulting in slight changes of the *Q*_10_ value by ~0.1 unit.

The temperature dependence of amplitudes often provides clues to elucidate oscillatory mechanisms behind and to construct models. Interestingly, the amplitude of PTO was reduced as the temperature decreased (Figure 4), while such temperature dependence was not obvious for that of TTO (Figure 6). In the case of cyanobacterial PTO, the decreased amplitude at lower temperatures is explained by a transition through the Hopf bifurcation [18]. The recent observation of TTO in mammalian cultured cells suggests a mechanism of temperature-amplitude coupling to explain its temperature-compensated period but temperature-sensitive amplitudes [19]. Although the reduced/enhanced amplitudes cannot be targeted directly by our ATPase-based in vitro screening, the temperature-dependent KaiC mutants screened by our protocol may constitute another library to search for mutants exhibiting an irregular temperature dependence of the PTO/TTO amplitudes.

**Figure 7:**
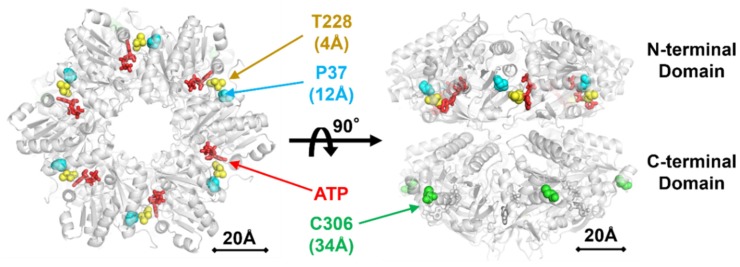
Three representative KaiC mutations (Pro37, Thr228, and Cys306) mapped on the crystal structure of wild-type KaiC carrying no alanine substitutions (accession code: 2GBL) [20]. Shown are distances to the nearest ATP in the N-terminal domain.

While two (KaiC-P37A and KaiC-T228A) of the three mutations characterized here reside in the N-terminal domain of KaiC, one (KaiC-C306A) locates in its C-terminal domain. The N-terminal domain of KaiC is the domain mostly responsible for ATPase [10,13]. According to the crystal structure of KaiC [20], Pro37 and Thr228 are close to an ATP molecule bound in the N-terminal domain with 12 and 4 Å distances, respectively (Figure 7). We infer from these observations that both KaiC-P37A and KaiC-T228A mutations directly affect the structure of the active site of ATPase in the N-terminal domain. On the other hand, Cys306 is distant from the active site by over 34 Å. The present screening is robust enough to identify a KaiC mutation affecting the ATPase activity indirectly through allosteric interactions, providing a practical means to uncover the regulatory mechanisms of ATPase in KaiC.

While the whole-KaiC alanine scanning is under way, there is every reason to be optimistic in screening the clock mutants of KaiC using the present method. This is because roughly half of the preliminary dataset thus far examined revealed the changes in the period length and/or its temperature-dependency (Figure 3a). Further modulations of the period length and/or its temperature-dependency can be expected by mutating a critical residue identified through the present protocol into residues other than alanine. The developed ATPase-based screening is a powerful tool for comprehensive mapping of the structural units regulating slow but temperature-compensated ATPase of KaiC.

## 4. Materials and Methods

### 4.1. Expressions and Purifications

Transformed *E. coli* cells were pre-cultured in 10 mL of Luria-Bertani (LB) broth medium containing 0.04 mg/mL ampicillin and 0.034 mg/mL chloramphenicol at 37 °C for 16 h and then transferred to 400 mL of terrific broth medium containing 0.04 mg/mL ampicillin. Bacterial growth was monitored with OD_600_ every 30 min prior to isopropyl β-d-thiogalactopyranoside (IPTG) induction. After the culture reached OD_600_ of 1.5 (approximately 3 h), over-expression of His-tagged KaiC was induced by adding IPTG to a final concentration of 0.1 mM. The cells were further harvested for approximately 5 h until OD_600_ reached ~6. The OD_600_ was measured after 10-fold dilution of the culture medium. The harvested cells were collected by centrifugation and then kept at −20 °C until use.

Multiple open columns filled with Ni-Sepharose resin and PD-10 (GE Healthcare, Chicago, IL, US) were used to purify multiple KaiC mutants simultaneously. Both KaiA and KaiB were expressed as GST-tagged forms and purified after the cleavage of the GST-tag, as described previously [21].

### 4.2. Development of Temperature-Controlled Sample Tables

An aluminum sample-holder produced to achieve the best fit to sample vials (Hitachi, Tokyo, Japan) was placed on an assembly of a Peltier device, a cooling fin, and a fan (Alpha, Shizuoka, Japan). The sample temperature in each sample table was monitored directly with a Pt100 sensor and controlled by a high-precision temperature controller (CELL System, Kanagawa, Japan).

### 4.3. ATPase Measurements

ATPase measurements were conducted routinely by using an HPLC system (EXTREMA, JASCO, Tokyo, Japan) equipped with the four sample tables each thermostated independently at 24, 27, 30, and 35 °C. ADP was separated from ATP on an Inert Sustain C18 column (2 μm, 3.0 × 50 mm) (GL Sciences, Tokyo, Japan) at a flow rate of 0.4 mL min^-1^. After the measurement, the ADP concentrations were calculated from their peak areas as reported previously [10,17]. ATPase activity was determined from a slope of a linear fit to time-evolution of the produced ADP. The activity was defined as the number of ADP produced by one KaiC molecule in a monomer unit per day.

### 4.4. KaiC Phosphorylation/Dephosphorylation Cycle

Phosphorylation/dephosphorylation cycles were reconstructed in vitro by mixing KaiC or its mutants with both KaiA and KaiB in the presence of ATP at 25, 30, and 35 °C [9]. A fraction of the phosphorylated KaiC (*f*_phos_) at each time point was analyzed by using sodium dodecyl sulfate-polyacrylamide gel electrophoresis (SDS-PAGE) [21] and *LOUPE* [22]. The period lengths were estimated by fitting *f*_phos_ values to the following equation:

(1) $$f_{\mathrm{phos}}=B+A\cos\left(2\pi \frac{t+\Phi }{P}\right)$$

where *A* is amplitude, *B* is baseline, *t* is incubation time, *Φ* is phase, and *P* is period. *Q*_10_ values were determined from the slope of an Arrhenius plot of cycle frequency as described previously [23].

### 4.5. In Vivo Bioluminsescence Assay

The bioluminescence assays were conducted as previously reported [7,9]. The KaiC open reading frame in pC*kaiABC* [7] was mutagenized using QuickChange II Site-Directed Mutagenesis Kits (Agilent Technologies, Santa Clara, CA, US) to change P37, T228, and C306. Cyanobacterial cells with the *kaiBC*-reporter cassette were pre-cultured for 3 days at 30 °C on BG-11 solid medium under constant light (LL) conditions [45 μE m^-2^ S^-1^ from light emitting diode daylight lamp]. After a dark treatment for 12 h, cells were then transferred to LL conditions at 25, 29, or 35 °C. The bioluminescence from the cells was recorded using a photomultiplier tube detector at 25, 29, and 35 °C.

## Figures and Tables

**Table 1:** Temperature dependency of ATPase activity of KaiC, period length of in vitro post-translational oscillations (PTO), and period length of in vivo transcriptional and translational oscillations (TTO).

KaiC	In Vitro ATPase Activity ^1,2^		In Vitro Phosphorylation Cycle ^1,3^		In Vivo Bioluminescence Rhythm ^1^	
	(d^−1^) ^4^	*Q*_10_	Period (h) ^4^	*Q*_10_	Period (h) ^5^	*Q*_10_^6^
WT	13.4 ± 0.9	1.01 ± 0.04	23.1 ± 0.3	1.09 ± 0.00	24.8 ± 0.2	1.05 ± 0.01 (0.99 ± 0.01)
P37A	12.9 ± 1.6 *	1.03 ± 0.02 *	24.6 ± 0.3 ***	1.08 ± 0.00 ***	28.1 ± 0.1 ****	1.01 ± 0.01 *** (0.93 ± 0.01 ***)
T228A	9.8 ± 0.9 ***	1.18 ± 0.06 **	30.2 ± 0.4 ****	1.15 ± 0.01 ***	30.2 ± 0.2 ****	1.17 ± 0.03 ** (0.94 ± 0.03 *)
C306A	15.9 ± 2.2 *	1.14 ± 0.05 **	19.6 ± 0.1 ****	1.14 ± 0.02 **	20.8 ± 0.0 ****	1.09 ± 0.01 *** (1.05 ± 0.01 ****)

^1^ Results are shown as the mean ± standard deviation from three independent preparations and measurements. ^2^ Steady-state ATPase activity of KaiC alone in the absence of both KaiA and KaiB. ^3^ Phosphorylation rhythms of KaiC and its mutants in the presence of both KaiA and KaiB. ^4^ Measurements at 30 °C. ^5^ Measurements at 29 °C. ^6^ Estimates using data taken at 25 and 29 °C. Values in parenthesis correspond to estimates using data taken at 25, 29, and 35 °C. T-tests against KaiC-WT: **** significant (*p* < 0.001), *** significant (*p* < 0.01), ** significant (*p* < 0.05), and * insignificant (*p* > 0.05).

## Abbreviations

HPLC	high-performance liquid chromatography
ATP	adenosine triphosphate
ADP	adenosine diphosphate
PTO	post-translational oscillation
TTO	transcriptional and translational oscillation
